# The effects of anesthesia methods and anesthetics on postoperative delirium in the elderly patients: A systematic review and network meta-analysis

**DOI:** 10.3389/fnagi.2022.935716

**Published:** 2022-11-03

**Authors:** Xuhui Zhuang, Yuewen He, Yurui Liu, Jingjing Li, Wuhua Ma

**Affiliations:** ^1^Department of Anesthesiology, The First Affiliated Hospital of Guangzhou University of Chinese Medicine, Guangzhou, Guangdong, China; ^2^Department of Anesthesiology, Jincheng People’s Hospital, Jincheng, China

**Keywords:** postoperative delirium, anesthesia, anesthetics, the older, network meta-analysis

## Abstract

**Study objective:**

Postoperative delirium (POD) is one of the serious postoperative complications in elderly patients, which is always related to long-term mortality. Anesthesia is often considered a risk factor for POD. This systematic review and network meta-analysis (NMA) aimed to assess the impact of different anesthesia methods and anesthetics on POD.

**Measurements:**

We searched for studies published in PubMed, Embase, Web of Science, Scopus, and Cochrane Library (CENTRAL) from inception to 18 March 2022. RevMan 5.3 and CINeMA 2.0.0 were used to assess the risk of bias and confidence. Data analysis using STATA 17.0 and R 4.1.2. STATA 17.0 was used to calculate the surface under the cumulative ranking curve (SUCRA) and provide network plots with CINeMA 2.0.0. NMA was performed with R 4.1.2 software gemtc packages in RStudio.

**Main results:**

This NMA included 19 RCTs with 5,406 patients. In the pairwise meta-analysis results, only regional anesthesia (RA) with general anesthesia (GA) vs. GA (Log OR: –1.08; 95% CI: –1.54, –0.63) were statistically different in POD incidence. In the NMA results, there was no statistical difference between anesthesia methods, and psoas compartment block (PCB) with bupivacaine was superior to the desflurane, propofol, sevoflurane, and spinal anesthesia with bupivacaine of POD occurrence.

**Conclusion:**

Our study indicated that RA and GA had no significant effect on POD, and there was no difference between anesthesia methods. Pairwise meta-analysis showed that, except for RA with GA vs. GA, the rest of the results were not statistically different. Besides, PCB with bupivacaine may benefit to reduce POD incidence.

**Systematic review registration:**

https://www.crd.york.ac.uk/prospero/dis play_record.php?ID=CRD42022319499, identifier PROSPERO 2022 CRD42022319499.

## Introduction

Delirium can be defined as “acute brain failure”, an acute neurocognitive disorder characterized by fluctuating disturbances in attention, perception, and cognitive function (Inouye et al., 2014; Mattison, 2020). Elderly age (≥ 65 years old) and surgical factors are the two most common triggers of delirium (Rudolph and Marcantonio, 2011). Delirium was previously considered to be a transient and self-limiting syndrome (AGS/NIA Delirium Conference Writing Group, Planning Committee and Faculty, 2015), and a growing body of studies (Koster et al., 2009; Saczynski et al., 2012; Goldberg et al., 2020) is now leading to an awareness of the long-term adverse effects of POD. It is significantly associated with higher perioperative and long-term mortality rates, prolonged hospital stays, long-term cognitive dysfunction, and other poor prognostic outcomes (Koster et al., 2012; Schnorr et al., 2022). Numerous physical and psychological complications will bring serious consequences to patients and a greater burden on the social healthcare system. It has been extrapolated that the cost of health care attributable to POD in the United States in 2021 is estimated at $32.9 billion, implying that POD is a large-scale public health problem (Gou et al., 2021).

The causes and potential mechanisms of delirium after major surgery are multiple, with the methods of anesthesia and anesthetics being potentially risk factors for POD. General anesthesia is usually the first choice for elderly patients undergoing major surgery, based on its safety, reliability, and convenience. However, it requires hypnotics, inhalational anesthetics, opioids, muscle relaxants, sedatives, and cardiovascular drugs to maintain a constant state of unconsciousness in the aged. Inhaled and intravenous anesthetics (Ölmeztürk Karakurt et al., 2022), benzodiazepines (Marcantonio et al., 1994; Rudolph and Marcantonio, 2011), and opioids (Dubois et al., 2001) are known or suspected risk factors for POD according to the available studies. Although perioperative opioid use is a risk factor for POD, it is difficult to avoid after major surgery because inadequate analgesia may increase the risk of POD (Morrison et al., 2003). In contrast, regional anesthetic methods, such as spinal and epidural anesthesia (EA), offer various potential advantages. Epidural anesthesia and analgesia are recommended and widely used for chest and abdominal surgeries (Chou et al., 2016). The benefits included continuous pain control, low opioid consumption, and reduction of patients’ stress and inflammatory response. Regional anesthesia has also reported that it can reduce the incidence of POD. However, several high-quality systematic reviews in recent years indicated that there were no significant differences in POD incidence when comparing general anesthesia with regional anesthesia (Bryson and Wyand, 2006; Guay et al., 2016).

There is currently no conclusive evidence that any anesthesia methods or anesthetics can prevent POD (Patel et al., 2018). A clear purpose and methodologically rigorous study to determine the effect of anesthesia methods and anesthetics for POD is warranted. So, it is concluded that this study extracted data from high-quality RCTs, and we designed this systematic review and network meta-analysis (NMA) to evaluate the effects of anesthesia methods and anesthetics on POD in elderly patients.

## Methods

### Study protocol

This systematic review and NMA have been registered with PROSPERO (registration number: CRD42022319499). And we followed the Systematic Reviews and meta-analysis (PRISMA) guidelines for this NMA.

### Search strategy

**FIGURE 1:**
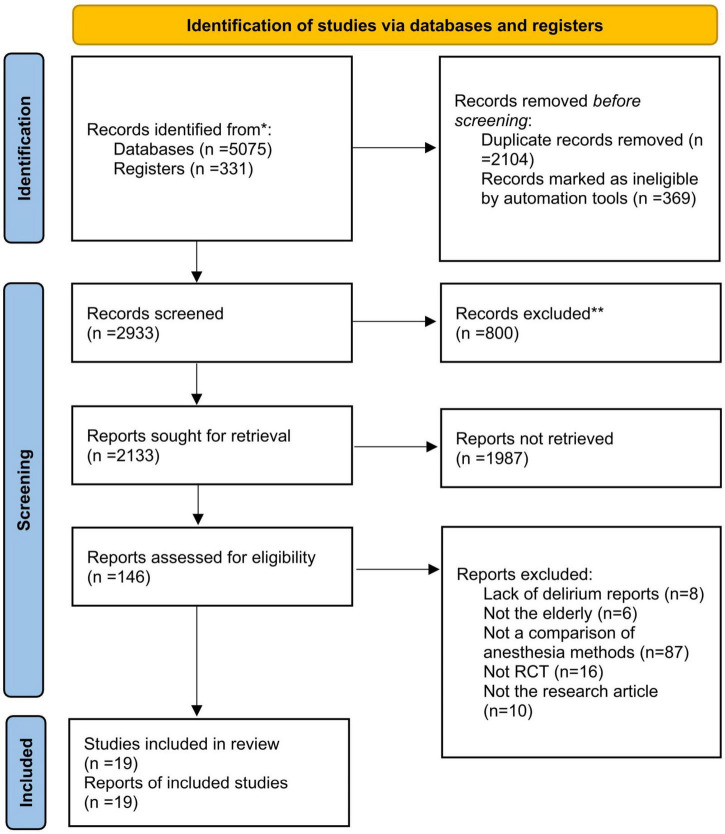
PRISMA flow diagram of the search strategy and included studies.

We searched for studies published in PubMed, Embase, Web of science, Scopus, and Cochrane Library (CENTRAL) from inception to 18 March 2022, without language restriction. We recorded the search process in PRISMA_2020_flow_diagram (Figure 1). The search strategy was designed based on inclusion and exclusion criteria (Table 1). All disputes were decided by W.M, who was not involved in the search. Before searching, two investigators identified keywords: delirium, postoperative, elderly, and anesthesia. The search terms for this NMA were determined by synonym queries and similar terms of key meta-analysis. Search algorithms included the following terms: (Delirium OR confusion OR disorientation OR acute confusional syndrome OR postoperative delirium) AND (postoperative OR perioperative OR operative OR operation OR surgery OR surgical) AND (Elderly Patients OR aged OR the aged OR old people OR the elderly OR elder OR agedness) AND (Anesthesia). We changed the search formula for different databases. For example, we also searched MeSH terms relevant to “Anesthesia” and “delirium”.

**TABLE 1:** The inclusion and exclusion criteria.

Inclusion criteria	Exclusion criteria
*Study type:* published RCTs, either single-blind or double-blind. *Language restriction:* No. *Participants:* the elderly population (age ≥ 65 years) with surgery. *Intervention:* regional anesthesia (spinal anesthesia, epidural anesthesia, nerve block), general anesthesia (intravenous anesthesia, volatile anesthesia), and both. *Outcomes:* incidence of new-onset delirium.	*Study type:* all of NRCTs or unpublished RCTs. *Participants:* not elderly patients (age < 65 years) and not undergoing surgery. *Intervention:* comparison of analgesic methods, preoperative and postoperative medication. *Outcomes:* no reports of delirium or not new onset.

RCT, Randomized Controlled Trial; NRCT, Non-Randomized Controlled Trial.

### Eligibility criteria

We set the inclusion criteria as follows: study type: published RCTs, either single-blind or double-blind; language restriction: no; participants: the elderly population (age ≥ 65 years) with surgery; intervention: regional anesthesia (spinal anesthesia, epidural anesthesia, nerve block), general anesthesia (intravenous anesthesia, volatile anesthesia), and both; outcomes: incidence of new-onset delirium.

Besides, we set the exclusion criteria as follows: study type: all of NRCTs or unpublished RCTs; participants: not elderly patients (age < 65 years) and not undergo surgery; intervention: Comparison of analgesic methods, preoperative and postoperative medication; outcomes: no reports of delirium or not new onset.

### Study selection

Two investigators (XZ and YH) used EndNote X9 (Thomson Reuters, NY, USA) to complete the study selection, and the process was divided into three parts. First, we excluded all duplicates and incomplete studies. Next, we initially reviewed the titles, keywords, and abstracts of all studies and graded them according to the inclusion criteria (low correlation, moderate correlation, and high correlation). During this process, studies were defined as low correlation, moderate correlation, and high correlation based on inclusion and exclusion criteria. When screening titles, abstracts, and keywords, if the research does not meet the inclusion criteria at all, it is defined as “low correlation”; “moderate correlation” means that most of the research contents meet the inclusion criteria, but some features are unclear and need to be reviewed again; “high correlation” means that the study fully meets the inclusion criteria. Second, we excluded all studies defined as “low correlation”. For “moderate correlation” studies, we reviewed the title, keywords, and abstract again. Finally, we reviewed the full text of the remaining studies defined as “moderate correlation”, as well as all studies with “high correlation”. All disputes during the study selection process were resolved by WM.

### Risk of bias assessment

The risk of bias of a single included RCT was analyzed in Review Manager 5.3 (RevMan, The Cochrane Collaboration, Oxford, United Kingdom) by two reviewers (XZ and YH), and divided into high risk, low risk, and unclear. This is based on the Cochrane Collaboration’s tool, which includes: selection bias, performance bias, detection bias, attrition bias, reporting bias, and other biases. In addition, we use Confidence in NMA (CINeMA 2.0.0 version) to analyze the confidence of the results. The following six factors can affect the confidence of NMA results: within-study bias, reporting bias, indirectness, imprecision, heterogeneity, and incoherence.

### Data extraction

Two investigators (XZ and YH) were responsible for data extraction for included studies independently, and all disputes were resolved by WM. We extracted the characteristics of the studies and patients and summarized them in Tables 2, 3. The contents are as follows: Author, year of publication, country, publications, study period, matched factors, ages, gender, and preoperative delirium are summarized in Table 2, and the characteristics of the anesthesia method are summarized in Table 3.

**TABLE 2:** Summary of the characteristics of patients in 19 eligible studies.

Author Yr, Country	Publications	Study period	Group (n)	Ages (Yr)	Gender (M/F)
Yoshida et al., 2008, Japan	Japan	ND	SA-lido(40)	Mean ± SD: 71 ± 6	ND
			GA-prop(40)	Mean ± SD: 73 ± 6	ND
Al Tmimi et al., 2020, Belgium	British Journal of Anesthesia	November 2015 to December 2017	GA-xenon(96)	Median (IQR): 76 (71–80)	53/43
			GA-sevo(94)	Median (IQR): 76 (70–81)	46/48
Dai et al., 2021, China	Medical Science Monitor	March 2016 to December 2017	GA-sevo(81)	Mean ± SD: 72 ± 7	76/5
			GA-prop(83)	Mean ± SD: 73 ± 8	74/9
Ukolov et al., 2020, Russia	Journal of Traumatology and Orthopedics	ND	SA-bupi(60)	Mean ± SD: 65.4 ± 6.5	ND
			SA-levobupi(30)	Mean ± SD: 65.5 ± 8.1	ND
Williams-Russo et al., 1995, USA	JAMA	October 1989 to October 1992	EA-lido or bupi(134)	Mean 69	63/71
			GA-prop(128)	Mean 69	58/70
Bielka et al., 2021, Ukraine	BMC Anesthesiology	January 2018 to August 2019	PCB-bupi(30)	Median (IQR): 72 (68–73)	21/9
			SA-bupi(30)	Median (IQR): 72 (70–73)	21/9
			GA-sevo(30)	Median (IQR): 73 (72–74)	22/8
Brown et al., 2021, USA	Anesthesiology	September 2015 to May 2019	SA-lido or bupi with sedation-prop(111)	Median (IQR): 73 (69–78)	48/63
			GA-prop(106)	Median (IQR): 72 (69–76)	35/71
Chen et al., 2002, USA	Anesthesia Analgesia	ND	GA-desf(35)	Mean ± SD: 75 ± 8	20/15
			GA-sevo(35)	Mean ± SD: 73 ± 9	18/17
Coburn et al., 2018, Germany	British Journal of Anesthesia	September 2010 to October 2014	GA-xenon(124)	Mean ± SD: 83.8 ± 5.1	34/90
			GA-sevo(132)	Mean ± SD: 84.4 ± 4.6	29/103
Gu et al., 2021, China	Clinical Journal of Pain	June 2019 to June 2020	NB-ropi with sedation-dex or prop(42)	Median (IQR): 74.5 (60–88)	19/23
			GA-prop(45)	Median (IQR): 69.3 (60–91)	18/27
Li et al., 2021, China	JAMA	October 2014 to September 2018	SA,EA or both(471)	Median (IQR): 77 (72–82)	128/343
			GA(471)	Median (IQR): 77 (71–82)	119/352
Li et al., 2021, China	Anesthesiology	November 2011 to May 2015	EA and GA-lido and ropi(857)	Mean ± SD: 69 ± 6	542/315
			GA-prop(863)	Mean ± SD: 70 ± 6	581/282
Liu et al., 2022, China	Medical Science Monitor	December 2020 to March 2021	TAPB and RSB-ropi and dex(50)	Mean ± SD: 70.50 ± 4.568	25/25
			Control(50)	Mean ± SD: 72.06 ± 5.266	26/24
Mei et al., 2020, China	Journal of Alzheimer’s Disease	June 2016 to November 2019	GA-sevo(103)	Mean ± SD: 71.5 ± 6.8	27/76
			GA-prop(106)	Mean ± SD: 70.9 ± 6.7	34/72
Neuman et al., 2021, USA	The New England Journal of Medicine	NR	SA-prop(795)	Mean ± SD: 77.7 ± 10.7	258/537
			GA-prop(805)	Mean ± SD: 78.4 ± 10.6	270/535
Shin et al., 2020, Korea	Journal of Clinical Medicine	May 2015 to January 2019	GA-desf(60)	Mean ± SD: 79.4 ± 7.7 13/47	
			GA-prop(58)	Mean ± SD: 80.5 ± 6.7	16/42
			SA-bupi(58)	Mean ± SD: 81.6 ± 6.7	17/41
Siripoonyothai and Sindhvananda, 2021, Thailand	Annals of Cardiac Anesthesia	June 2019 to February 2020	GA-keta(32)	ND	14/18
			GA-prop(32)	ND	21/11
Tanaka et al., 2017, USA	Journal of Clinical Anesthesia	October 2010 to August 2014	GA-desf(45)	Median (IQR): 69.8(68.6–71.1)	25/20
			GA-prop(45)	Median (IQR): 70.6(69.2–72.1)	15/30
Tang et al., 2021, China	Evidence-Based Complementary and Alternative Medicine	August 2019 to December 2019	GA with CLSPB-ropi(55)	Mean ± SD: 76.60 ± 6.98	20/35
			SA-ropi with sedation-prop(55)	Mean ± SD: 78.00 ± 6.45	16/39

ND, not declared; Yr, year; M, male; F, female; SD, standard deviation; IQR, interquartile range; SA, spinal anesthesia; EA, epidural anesthesia; GA, general anesthesia; NB, nerve block; PCB, psoas compartment block; TAPB, transversus abdominis plane block; RSB, rectus sheath block; CLSPB, combined lumbar and sacral plexus block; lido, lidocaine; bupi, bupivacaine; levobupi, levobupivacaine; ropi, ropivacaine; prop, propofol; dex, dexmedetomidine; keta, katamine; sevo, sevoflurane; desf, desflurane.

**TABLE 3:** Summary of the anesthesia methods in the 19 eligible studies.

Author, Yr	Country	Surgery	Group	Intervention
Yoshida et al., 2008	Japan	prostate biopsy	Group1: SA-lido	1% lidocaine
			Group2: GA-prop	Induction: propofol 1mg/kg Maintenance: propofol 8mg/kg/h
Al Tmimi et al., 2020	Belgium	on-pump cardiac surgery	Group1: GA-xenon	Induction: remifentanil, propofol, cisatracurium Maintenance: xenon 40-60%
			Group2: GA-sevo	Induction: remifentanil, propofol, cisatracurium Maintenance: sevoflurane 1.0-1.4%
Dai et al., 2021	China	major non-cardiac surgery with CAD	Group1: GA-sevo	Induction: fentanyl, etomidate, cisatracurium Maintenance: sevoflurane-remifentanil
			Group2: GA-prop	Induction: fentanyl, etomidate, cisatracurium Maintenance: propofol-remifentanil
Ukolov et al., 2020	Russia	knee and hip arthroplasty	Group1: SA-bupi	0.5% bupivacaine
			Group2: SA-levobupi	0.5% levobupivacaine
Williams-Russo et al., 1995	USA	total knee replacement	Group1: EA-lido or bupi	2% lidocaine or 0.75% bupivicaine Sedation: midazolam and fentanyl.
			Group2: GA	Induction: thiopental, sodium, fentanyl, vecuronium Maintenance: fentanyl, nitrous oxide, isoflurane
Bielka et al., 2021	Ukraine	osteosynthesis of the proximal femur	Group1: PCB-bupi	bupivacaine Sedation: propofol
			Group2: SA-bupi	bupivacaine Sedation: propofol
			Group3: GA-sevo	sevoflurane
Brown et al., 2021	USA	lumbar spine fusion	Group1: SA-lido or bupi with sedation-prop	bupivacaine or lidocaine. Sedation: propofol
			Group2: GA	Induction: propofol or etomidate Maintenance: volatile anesthetic, non-depolarizing muscle relaxant
Chen et al., 2002	USA	total knee or hip replacement	Group1: GA-desf	Induction: fentanyl, propofol, succinylcholine Maintenance: desflurane 2–4%, N2O 65% in oxygen
			Group2: GA-sevo	Induction: fentanyl, propofol, succinylcholine Maintenance: sevoflurane 1.0–1.5%, N2O 65% in oxygen
Coburn et al., 2018	Germany	hip fracture	Group1: GA-xenon	5% xenon in oxygen (FiO2 = 0.35 to 0.45)
			Group2: GA-sevo	1.1 –1.4% sevoflurane in oxygen (FiO2 = 0.35 to 0.45)
Gu et al., 2021	China	hip fracture	Group1: NB-ropi with sedation-dex or prof	0.3% ropivacaine Sedation: dexmedetomidine, propofol
			Group2: GA	Induction: propofol, rocuronium bromide, sufentanyl Maintenance: propofol, sevoflurane
Li et al., 2021	China	hip fracture	Group1: SA, EA or both	ND
			Group2: GA	ND
Li et al., 2021	China	major non-cardiac thoracic or abdominal surgery	Group1: EA and GA-lido and ropi	lidocaine and ropivacaine during surgery
			Group2: GA	Induction: midazolam, propofol, sufentanil and rocuronium Maintenance: propofol, sevoflurane, and nitrous oxide
Liu et al., 2022	China	laparoscopic colorectal cancer radical surgery	Group1: TAPB and RSB-ropi and dex	ropivacaine, dexmedetomidine GA: Induction: fentanyl, etomidate, cis-atracurium Maintenance: propofol, remifentanil and cisatracurium
			Group2: Control	Induction: fentanyl, etomidate, cis-atracurium Maintenance: propofol, remifentanil and cisatracurium
Mei et al., 2020	China	total hip or knee replacement	Group1: GA-sevo	sevoflurane
			Group2: GA-prop	propofol
Neuman et al., 2021	USA	hip fracture	Group1: SA	ND
			Group2: GA	ND
Shin et al., 2020	Korea	hip fracture	Group1: GA-desf	Induction: pentothal sodium, cisatracurium, and remifentanil Maintenance: desflurane, remifentanil
			Group2: GA-prop	Induction: propofol, remifentanil, and cisatracurium Maintenance: propofol, remifentanil
			Group3: SA-bupi	bupivacaine Sedation: midazolam
Siripoonyothai and Sindhvananda, 2021	Thailand	cardiac surgery with CPB	Group1: GA-keta	ketamine, fentanyl and cisatracurium
			Group2: GA-prop	propofol, fentanyl and cisatracurium
Tanaka et al., 2017	USA	total knee replacement	Group1: GA-desf	Induction: propofol, fentanyl and rocuronium Maintenance: desflurane Femoral nerve block: ropivacaine Sedation: fentanyl and midazolam
			Group2: GA-prop	Induction: propofol, fentanyl and rocuronium Maintenance: propofol Femoral nerve block: ropivacaine Sedation: fentanyl and midazolam
Tang et al., 2021	China	osteosynthesis, artificial femoral head replacement and total hip replacement	Group1: GA with CLSPB-ropi	GA: propofol, sufentanil and cisatracurium CLSPB: ropivacaine
			Group2: SA-ropi with sedation-prop	SA: ropivacaine Sedation: propofol

ND, not declared; Yr, year; SA, spinal anesthesia; EA, epidural anesthesia; GA, general anesthesia; NB, nerve block; PCB, psoas compartment block; TAPB, transversus abdominis plane block; RSB, rectus sheath block; CLSPB, combined lumbar and sacral plexus block; lido, lidocaine; bupi, bupivacaine; levobupi, levobupivacaine; ropi, ropivacaine; prop, propofol; dex, dexmedetomidine; keta, katamine; sevo, sevoflurane; desf, desflurane; CAD, coronary heart disease CPB, cardiopulmonary bypass.

### Outcomes

We included RCTs to assess the effect of anesthesia modality on the incidence of POD in the elderly. The primary outcome of this NMA is the incidence of POD in elderly patients with different anesthesia methods or anesthetics. Delirium was diagnosed by several tools as follows: Confusion Assessment Method (CAM), Delirium Rating Scale-Revised-98, and Short Portable Mental Status Questionnaire. Secondary outcomes include the occurrence of postoperative nausea and vomiting (PONV) and hypotension. Hypotension was defined as systolic blood pressure (SBP) < 90 mm Hg or more than 20% reduction compared to preoperatively.

### Statistical analysis

First, STATA (version 17.0) was used to perform a conventional pair-wise meta-analysis of direct evidence. The outcomes of this study were all dichotomous, and we calculated the odds ratio (OR) with 95% confidence intervals (CIs) in the random effect model. In addition, we used CINeMA 2.0.0 and STATA 17.0 to generate network plots for different groups, which visualized the relationship between various interventions. The size of the node in the network plot represents the sample size of the group, and the color is the risk of bias (Green: low risk; Yellow: unclear; Red: high risk). The edge width represents the number of studies.

Second, NMA was performed with R 4.1.2 software gemtc packages in RStudio, based on the Bayesian framework with Markov Chain Monte Carlo simulation. We ran the estimation with a burn-in of 25,000 iterations and a sampling of 50,000 iterations from four chains of initial values. The selection between models is based on deviance Information Criteria (DIC). If the DIC difference in the consistency test results is greater than 5, the difference is considered to be significant. The fluctuation process of the MCMC chain is represented by the trace plot, and the convergence degree of the model is diagnosed together with the density plot.

Third, we used STATA 17.0 to calculate the surface under the cumulative ranking (SUCRA) to rank the interventions. For a given intervention, the larger value of the SUCRA, the more significant of effect in this ranking. For the analysis results of this study, two-tailed tests with *P* < 0.05 was defined as statistically significant.

## Results

We searched five databases with a total of 5,406 articles and screened them according to inclusion and exclusion criteria. The screening procedure is described in the Study Selection section of Methods. In the first part, we excluded 2,104 duplicate studies and 1,169 irrelevant studies. In the second part, we screened the titles and abstracts of the included studies, excluded all studies with “low correlation”, and performed a secondary screening of studies defined as “moderate correlation,” excluding 1,987 studies. Finally, we conducted a full-text review of 146 studies, and 19 RCTs were included in this systematic review and NMA. The search process is represented in PRISMA_2020_flow_diagram (Figure 1).

### Study and patient characteristics

In 19 RCTs, 5,406 patients were included in this NMA. The investigators extracted the characteristics of the patients in the 19 studies, containing authors, countries, publications, study period, ages (Years), and gender in Table 2. The data of ages were reported using Mean (Standard Deviation, SD) or Median (Inter Quartile Range, IQR). In the study of Williams-Russo et al. (1995), only the mean age was described for the included patients. Moreover, Siripoonyothai and Sindhvananda (2021) did not describe the age of the included patients. Two studies were divided into three groups for comparison. Bielka et al. (2021) chose to use two different RA_bupi to compare with GA_sevo. And among the study of Shin et al. (2020), the patients were randomly divided into GA_desf, GA_prop, and SA groups.

### Intervention characteristics

The researchers extracted intervention characteristics of included studies, which mainly focused on the type of surgeries, the groups, and the interventions (Table 3). Six studies (Coburn et al., 2018; Shin et al., 2020; Gu et al., 2021; Neuman et al., 2021; Tang et al., 2021; Li et al., 2022) clearly stated that the data came from the elderly who had undergone hip fracture surgery. And three studies reported that the patients underwent total knee or hip replacement surgery. Only Al Tmimi et al. (2020) and Siripoonyothai and Sindhvananda (2021) included patients from cardiac surgery. All but five studies (Coburn et al., 2018; Mei et al., 2020; Bielka et al., 2021; Neuman et al., 2021; Li et al., 2022) did not report or did not use fentanyl-type analgesics during the anesthesia induction or maintenance phases.

### Risk of bias assessment and study confidence rating

We assessed the risk of bias for 19 RCTs using RevMan 5.3. The studies by Yoshida et al. (2008) and Ukolov et al. (2020) lacked descriptions of random sequence generation and concealment, which were defined as “unclear.” Williams-Russo et al. (1995) also did not indicate allocation concealment. The study defined unblinded or single-blind studies as high risk, and only three studies (Chen et al., 2001; Tanaka et al., 2017; Coburn et al., 2018) defined as low risk were double-blind. In addition, Williams-Russo et al. (1995) were not blinded to the outcome assessment (Supplementary Figure 1).

CINeMA 2.0.0 was used to assess the confidence of included studies. We analyzed the delirium data of the two groups in anesthesia methods and anesthetics, respectively. In delirium in anesthesia methods, there are three comparisons defined as moderate confidence ratings because of major concerns in imprecision (Supplementary Figure 2). In the group of delirium in anesthetics, six comparisons are defined as moderate confidence ratings due to within-study bias, heterogeneity, imprecision, and indirectness. Besides, there is a comparison defined as a low confidence rating due to major concerns about within-study bias and imprecision (Supplementary Figure 3). STATA 17.0 was used to assess publication bias in primary outcomes’ comparisons, including the occurrence of POD across different anesthesia, anesthesia methods, and anesthetics. Funnel plots are provided in Supplementary Figures 4–6.

### Network plot of eligible comparisons of outcomes

**FIGURE 2:**
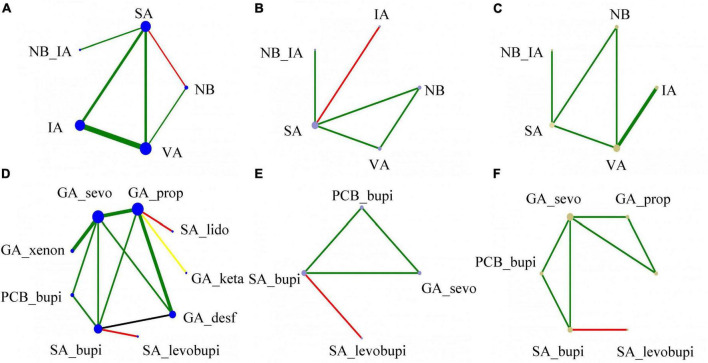
The network geometry (the incidence of POD) using colored edges according to the risk of bias (Green: low risk; Yellow: unclear; Red: high risk). **(A)** Delirium occurrence of anesthesia methods; **(B)** hypotension of anesthesia methods; **(C)** PONV occurrence of anesthesia methods; **(D)** delirium occurrence of different anesthetics; **(E)** hypotension of different anesthetics; **(F)** PONV occurrence of anesthetics. The size of the node in the network plot represents the sample size of the group, the edge width represents the number of studies, and the color is the risk of bias.

Figure 2 summarizes the network diagram of the primary and secondary outcomes. As described in the Statistical Analysis section in methods, the size of the network plot nodes represents the sample size, the width of the lines represents the number of included studies, and the color represents the risk of bias. Unlike the study by Cui et al. (2020), we did not refer to blinding alone as a source of risk of bias assessment. Following the risk of bias results described in Supplementary Figure 1, we quantified the risk of bias for each study as grades 1, 2, and 3. The quantification rules are as follows: the total score of the study < 3 is grade 1; score = 3 or 4 is grade 2; score > 4 is grade 3, of which high risk: 2 score, unclear: 1 score, low risk: 0 score. STATA 17.0 was used to generate Figure 2, and we referred to the plots generated by CINeMA 2.0.0 to optimize them.

### Consistency and heterogeneity results

The consistency and heterogeneity results are summarized in Table 4. As shown in the Methods section, the researchers built a consistency model to analyze outcomes based on the Bayesian framework in the random effect model. Previously, we detected global inconsistencies across all outcomes and compared results with consistent models. For all the outcomes data, the two models fit well and are consistent with the assumption of consistency (DIC difference < 5). In addition, the Node-Splitting method is used to evaluate the local inconsistency of the data. In the results of the primary outcomes, the local inconsistency of the data in the Anesthesia group was statistically significant (*P* < 0.05). To maintain the reliability of the conclusions, we only used direct meta-analysis comparisons for the data in the Anesthesia group, discarding the conservative NMA conclusions. The results of heterogeneity are also shown in Table 4. There was no significant heterogeneity in all data except for the direct comparison of RA with GA vs. RA in the Anesthesia group.

**TABLE 4:** Results of consistency and heterogeneity.

Treatment	Direct		Indirect		Network		P-value	I^2^ statistic
			
	OR	95% CI	OR	95% CI	OR	95% CI		
**Anesthesia**								
RA vs. GA	1.20	0.84–1.70	0.23	0.06–0.89	1.10	0.69–1.50	0.02	0.00
RA with GA vs. GA	0.34	0.19–0.56	1.60	0.46–6.30	0.42	0.25–0.74	0.03	0.00
RA with GA vs. RA	1.40	0.40–5.00	0.29	0.15–0.54	0.39	0.22–0.79	0.03	0.83
**Anesthesia methods**								
SA vs. IA	0.97	0.19–4.90	0.87	0.02–42.00	0.90	0.27–3.10	0.96	0.00
VA vs. IA	0.91	0.46–2.00	<0.01	out of range	0.91	0.48–1.90	0.62	0.00
VA vs. SA	1.1	0.27–4.40	>1.00	out of range	1.00	0.32	3.50	0.00
**Anesthetics**								
GA_prop vs. GA_desf	0.78	0.21–2.70	<0.01	out of range	0.78	0.22–2.60	0.69	0.00
GA_sevo vs. GA_desf	< 0.01	out of range	0.66	0.15–2.70	0.64	0.15–2.60	0.62	0.00
SA_bupi vs. GA_desf	0.90	0.19–4.10	0.34	0.00–35.00	0.81	0.20–3.10	0.68	0.00
GA_sevo vs. GA_prop	0.82	0.34–2.00	0.97	0.02–50.00	0.82	0.36–1.90	0.93	0.00
SA_bupi vs. GA_prop	0.99	0.21–4.80	0.90	0.02–41.00	1.00	0.28–3.90	0.96	0.00
SA_bupi vs. GA_sevo	0.99	0.02–43.00	1.30	0.24–7.40	1.30	0.30–5.40	0.88	0.00
GA_xenon vs. GA_sevo	NE	NE	NE	NE	NE	NE	NE	0.27

OR, odd ratio; CI, confidence interval; NE, Not estimate; IA, intravenous anesthesia; VA, ventilate anesthesia; SA, spinal anesthesia; NB, nerve block; GA, general anesthesia; SA, spinal anesthesia; CLSPB, Combined Lumbar-Sacral Plexus Block; PCB, Psoas compartment block; sevo, sevoflurane; prop, propofol; desf, desflurane; lido, lidocaine; bupi, bupivacaine; ropi, ropivacaine; keta, ketamine; I^2^ statistic, Heterogeneity evaluation indicator.

### Pairwise meta-analysis of outcomes

The pairwise meta-analysis results and heterogeneity of the primary outcome are listed in Tables 5, 6. It mainly reported the number of studies included in the comparison, OR, 95% CI, *P*-value, and the results of the heterogeneity test *I*^2^ statistic. Among the results of the different anesthesia methods and anesthetics groups, none of the comparisons were statistically significant. Furthermore, the results of the heterogeneity test were acceptable.

**TABLE 5:** The direct evidence from pairwise meta-analysis and heterogeneity of different anesthesia methods.

Treatment	Number of study	OR	95% CI	P-value	I^2^ statistic
SA vs. IA	2	1.00	0.35–2.87	1.00	NE
SA vs. VA	2	0.92	0.35–2.41	0.86	0.00
VA vs. IA	4	0.88	0.59–1.30	0.51	0.00
NB vs. VA	1	0.31	0.01–7.96	0.48	NE
NB vs. SA	1	0.31	0.01–7.96	0.48	NE
NB with IA vs. SA	1	1.39	0.45–4.31	0.57	NE

OR, odd ratio; CI, confidence interval; NE, Not estimate; IA, intravenous anesthesia; VA, ventilate anesthesia; SA, spinal anesthesia; NB, nerve block; I^2^ statistic, Heterogeneity evaluation indicator.

**TABLE 6:** The direct evidence from pairwise meta-analysis and heterogeneity of different anesthetics.

Treatment	Number of study	OR	95% CI	P-value	I^2^ statistic
1 vs. 2	2	0.82	0.53–1.26	0.36	0.41
3 vs. 1	2	0.92	0.59–1.42	0.70	0.25
4 vs. 2	2	1.22	0.47–3.13	0.68	0.00
5 vs. 2	1	NE	NE	NE	NE
2 vs. 6	1	1.00	0.35–2.87	1.00	NE
7 vs. 8	1	1.39	0.45–4.31	0.57	NE
6 vs. 9	1	NE	NE	NE	NE
10 vs. 1	1	0.31	0.01–7.96	0.48	NE
1 vs. 6	1	1.00	0.06–16.79	1.00	NE
10 vs. 6	1	0.31	0.01–7.96	0.48	NE
4 vs. 1	1	NE	NE	NE	NE
4 vs. 6	1	1.10	0.39–3.09	0.85	NE
11 vs. 2	1	0.35	0.13–0.98	0.05	NE

1: GA_sevo; 2: GA_prop; 3. GA_xenon; 4: GA_desf; 5: SA_lido; 6: SA_bupi; 7: CLSPB_ropi; 8: SA_ropi; 9: SA_levobupi; 10: PCB_bupi; 11: GA_keta. OR, odd ratio; CI, confidence interval; NE, Not estimate; GA, general anesthesia; SA, spinal anesthesia; CLSPB, Combined Lumbar-Sacral Plexus Block; PCB, Psoas compartment block; sevo, sevoflurane; prop, propofol; desf, desflurane; lido, lidocaine; bupi, bupivacaine; ropi, ropivacaine; keta, ketamine; I^2^ statistic, Heterogeneity evaluation indicator.

**FIGURE 3:**
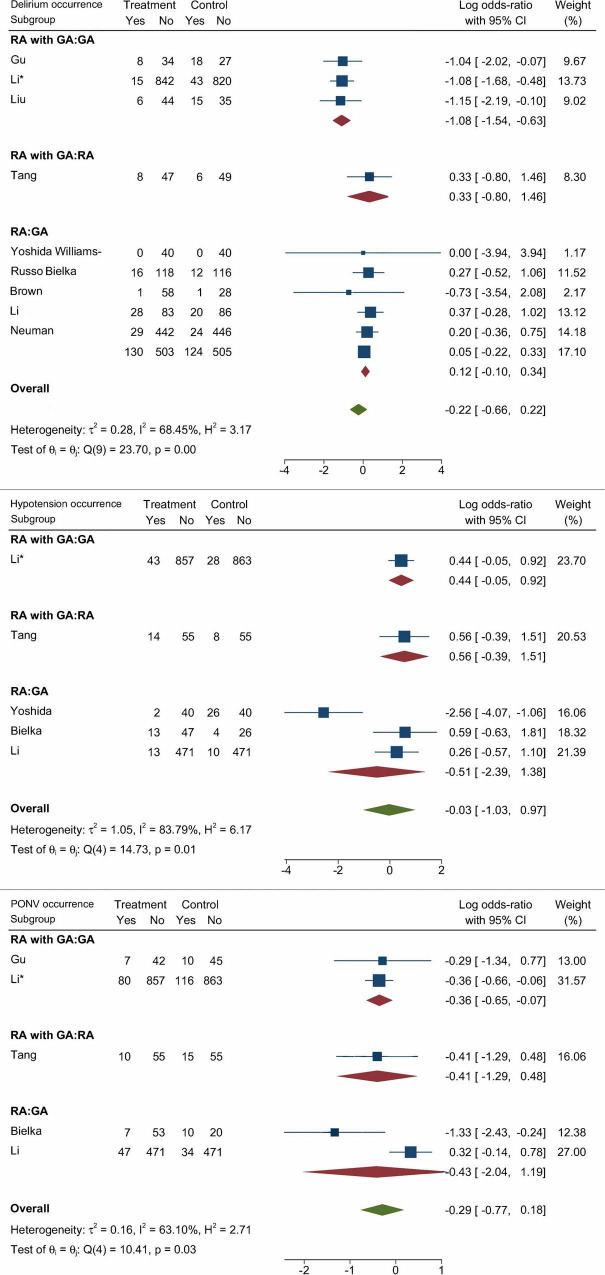
Pairwise meta-analysis results of the occurrence of POD, postoperative hypotension, and PONV (Data are expressed as Log OR, 95%CI).

The pairwise meta-analysis results of the Anesthesia group are shown in Figure 3. The forest plot provided outcomes, subgroup information, Log OR with 95% CI, and weights. The occurrence of POD, hypotension, and PONV were included in the outcomes. Of all the results, only the RA with GA vs. GA subgroup was statistically significant in the occurrence of POD (Log OR: –1.08; 95% CI: –1.54, –0.63) and PONV (Log OR: –0.36; 95% CI: –0.65, –0.07). However, the overall effect of the three groups of data was without statistically significant differences.

### Network meta-analysis of primary outcomes

**FIGURE 4:**
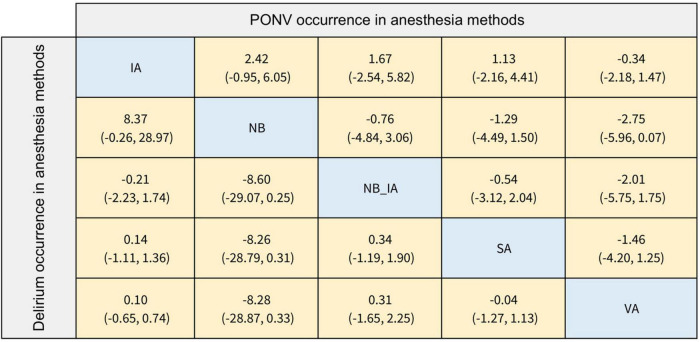
The league table of delirium and PONV occurrence in anesthesia methods (Data are expressed as Log OR, 95%CI).

The primary outcome of our NMA was the occurrence of POD with different anesthesia methods and anesthetics. The results of POD occurrence of different anesthesia methods are listed in Figure 4 (Log OR, 95% CI). Based on the included studies, we summarized five anesthesia methods in IA, NB, NB_IA, SA, and VA, and established comparisons and the NMA model. In terms of the occurrence of POD, none of the comparisons between any of the two anesthesia methods were statistically significant. In addition, as shown in Supplementary Figure 7, the SUCRA values of five anesthesia methods for POD occurrence were NB (79.1%), VA (53.3%), SA (51.0%), IA (37.1%), and NB_IA (29.4%).

**FIGURE 5:**
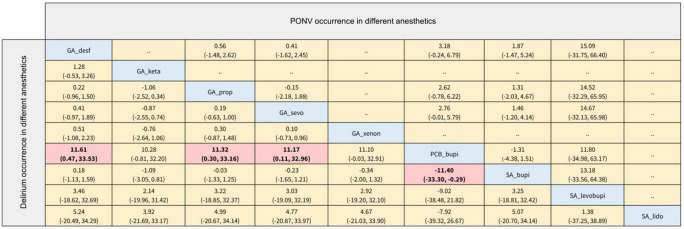
The league table of delirium and PONV occurrence in different anesthetics (Data are expressed as Log OR, 95%CI).

The results of the POD occurrence of different anesthetics are provided in Figure 5. We summarized nine anesthetics as follows, GA_sevo, GA_prop, GA_xenon, GA_desf, SA_lido, SA_bupi, SA_levobupi, PCB_bupi, and GA_keta. Compared with the Psoas compartment block with bupivacaine (PCB_bupi), general anesthesia with desflurane (GA_desf; Log OR: 11.61; 95% CI: 0.47, 33.53), general anesthesia with propofol (GA_prop; Log OR: 11.32; 95% CI: 0.30, 33.16), general anesthesia with sevoflurane (GA_sevo; Log OR: 11.17; 95% CI: 0.11, 32.96), and spinal anesthesia with bupivacaine (SA_bupi; Log OR: 11.40; 95% CI: 0.29, 33.30) were associated with a higher incidence of POD. The SUCRA values provided a hierarchy of nine treatments; 50.3%, 36.5%, 55.7%, 31.7%, 45.9%, 40.7%, 33.1%, 73.6%, 82.6% for GA_sevo, GA_prop, GA_xenon, GA_desf, SA_lido, SA_bupi, SA_levobupi, PCB_bupi, GA_keta, respectively. However, in ranking probability plots (Supplementary Figure 8), PCB_bupi might be a better choice (42.2%).

### Network meta-analysis of secondary outcomes

**FIGURE 6:**
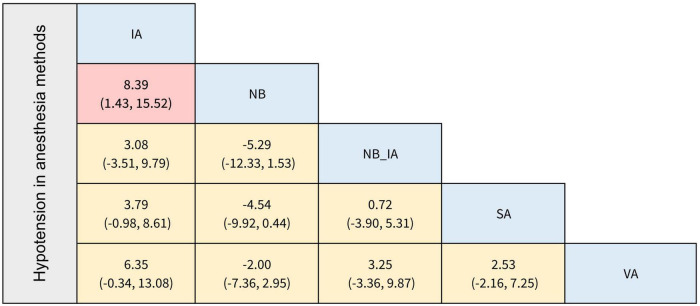
The league table of incidence of postoperative hypotension in anesthesia methods (Data are expressed as Log OR, 95%CI).

**FIGURE 7:**
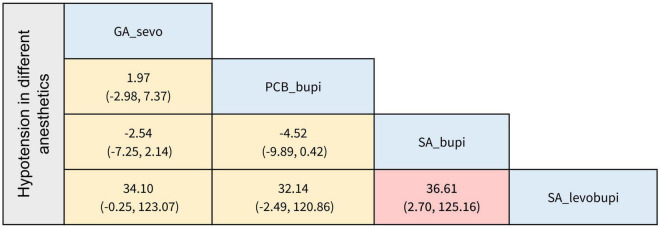
The league table of incidence of postoperative hypotension in different anesthetics (Data are expressed as Log OR, 95%CI).

The secondary outcomes of this NMA were the incidence of PONV and postoperative hypotension with different anesthesia methods and anesthetics. In terms of the occurrence of PONV listed in Figures 4, 5, none of the comparisons between any of the two anesthesia methods or anesthetics were statistically significant. The comparisons of postoperative hypotension occurrence for different anesthesia methods and anesthetics are summarized in Figures 6, 7. For the comparison of anesthesia methods, NB was more effective than IA in reducing postoperative hypotension (Log OR: 8.39; 95% CI:1.43, 15.52). In addition, SA_levobupi was superior to SA_bupi in reducing postoperative hypotension in anesthetics comparison (Log OR: 36.61; 95% CI:2.70, 125.16).

## Discussion

How to reduce the incidence of POD in the elderly population has always been a thorny issue that anesthesiologists seek to address. It is known that the consequences of POD range from minor disruptions to serious physical harm, such as catheter dislocation, prolonged hospital stay, long-term postoperative cognitive dysfunction, and high 1-year mortality (Pisani et al., 2009; Shehabi et al., 2010). In recent years, a large number of high-quality RCTs have emerged to compare the effects of different anesthesia methods or various anesthetics on POD. However, their different interventions led to various conclusions that prevented readers from finding the best option.

Our systematic review and network meta-analysis enrolled 19 RCTs involving 5,406 patients to evaluate the effects of different anesthesia methods and anesthetics on POD in older patients. Unlike the classical meta-analysis, we unfolded pairwise meta-analysis and network meta-analysis of different anesthesia methods with anesthetics. By performing a pairwise meta-analysis to compare different methods of anesthesia, we found that only GA combined with RA was statistically significant in reducing the incidence of POD when compared to GA. In the NMA stage, there was no statistical difference between anesthesia methods. Besides, PCB with bupivacaine may benefit to reduce POD incidence.

It has been widely reported that anesthesia is one of the major risk factors for POD in older people. The effect of GA versus RA on POD remains controversial. The general thought is that GA will cause more impairing to the cognitive function of the aged than RA (Chan et al., 2013; Evered and Silbert, 2018), which covers a range of intradural or peripheral nerve interventions, blocking spinal and/or EA, with or without nerve blocks. The relationship between GA and delirium is complex, and the mechanisms are not fully elucidated, but certainly, the need for multiple anesthesia drugs to maintain intraoperative sedation during GA would be more prone to POD than RA. Because RA does not require as much sedation as GA due to the different types of surgeries and shorter duration of the surgery. Intraoperative depth of sedation is thought to affect cognitive function, the shallower the depth of anesthesia, the lower the incidence of POD (Sieber et al., 2019). The relationship between perioperative depth of anesthesia and POD was determined by a prospective observational study (Soehle et al., 2015) that included 81 patients (age > 60 years old) undergoing cardiac surgery, with excessive depth of anesthesia being associated with an increased risk of POD related. Several meta-analyses conducted in recent years have exposed that over depth of anesthesia would relate to a greater risk of POD (MacKenzie et al., 2018; Li and Zhang, 2020). Opioid use may be another issue that can cause differences between GA and RA in terms of affecting POD. Numerous studies have revealed that medication, especially opioid analgesic, is a contributing factor to POD (Marcantonio et al., 1994; Weinstein et al., 2018). GA requires basic sedation and analgesic drugs to maintain a stable state during surgery, however, the opioid analgesic is a widely used drug for perioperative anesthesia management (Woods et al., 2021). Perioperative pain is also an influential contributor to POD (Vaurio et al., 2006; Rengel et al., 2018), and since opioids are often used for intraoperative pain control in older patients, both over- and under-analgesia can trigger POD. This makes it difficult to avoid the use of opioids in older people during GA. Along with reducing the need for sedative-hypnotic drugs with RA techniques, peripheral nerve and spinal anesthesia greatly reduce the need for opioid analgesics and their associated side effects, as well as provide high-quality analgesia.

However, Ellard et al. (2014) found that there was no statistical difference in the occurrence of POD between GA, RA, and local anesthesia (LA) in patients undergoing vascular surgery. The results of our pairwise meta-analysis also showed that RA had no benefits over GA in reducing POD for elderly patients. A multicenter RCT evaluated the effect of RA compared with GA on the incidence of POD in elderly patients undergoing hip fracture surgery. After including 950 patients (age ≥ 65 years old), Li et al. (2022) concluded that RA without sedation did not successfully reduce POD compared with GA. Likewise, Neuman et al. (2021) found a similar incidence of POD between SA and GA. Tang et al. (2021) compared the effect of combined lumbar-sacral plexus block plus GA with SA on the occurrence of POD in elderly populations undergoing hip fracture surgery and found no preferable. The explanation for why there is no difference between RA and GA in reducing POD is complicated by this result. Based on the speculation of Guay et al. (2016), the type of anesthesia methods used in many studies may not reflect current clinical practice, and RA still combines with sedatives or inhalation anesthetics, which may reduce the difference between the two anesthesia techniques. Perhaps it is since sedative drugs are still used adjunctively during RA or some deeper mechanisms that we need larger, more convincing RCTs to further validate.

The impact of one included RCT led to our outcomes showing that RA in combination with GA can markedly reduce the likelihood of POD in elderly patients more than GA alone. Li et al. (2021) designed an RCT including 1,802 patients who were randomly assigned to receive epidural-general anesthesia combined with postoperative epidural analgesia or GA combined with postoperative intravenous analgesia. The conclusion validated the hypothesis that epidural-general anesthesia reduced the incidence of POD in elderly patients recovering from major non-cardiac surgery, but with a risk of combined hypotension. The RA_GA can relieve surgical stress and reduce the need for volatile anesthetics and opioids, in addition to compensating GA for an incomplete blockade and inadequate muscle relaxation (Xu et al., 2021). Furthermore, due to its obvious advantages, Li et al. (2018) found that RA_GA had a lower risk of postoperative complications than GA in patients undergoing open surgery for pheochromocytoma.

Maintenance of GA or sedation state under RA requires the action of intravenous anesthetics and/or inhaled anesthetics. Sevoflurane and desflurane are inhaled anesthetics commonly used in VA owing to their low blood gas solubility coefficients. Previous studies have shown that volatile anesthetics may induce or exacerbate neuroinflammation, and are neurotoxic, such as by causing beta-amyloid deposition (Xie et al., 2008). Propofol has been used worldwide for safe and controlled IA, which may also induce neuronal cell death in the developing rat brain (Pesić et al., 2009). To compare the effects of IA and VA on the occurrence of postoperative neurocognitive adverse events in the elderly undergoing non-cardiac surgery, Tanaka et al. (2017) and Dai et al. (2021) designed RCTs comparing sevoflurane with propofol and desflurane with propofol, respectively. No differences were observed in POD incidence and early cognitive outcomes between the two groups. Xenon is a novel popular volatile anesthetic in the operating room in recent years. With an extremely low blood gas coefficient (0.115) (Nair et al., 2021), it has a fast onset of action and quick recovery in VA. Yet, several large RCTs (Coburn et al., 2018; Al Tmimi et al., 2020) reported no difference between xenon and volatile anesthetics concerning the risk of delirium after cardiac surgery or hip fracture surgery. CA meta-analysis conducted by Miller et al. (2018) showed that it could not be determined whether the use of propofol-based or inhaled anesthetics for GA would affect the incidence of POD, which is similar to our NMA results.

There is consistent evidence that RA can provide more effective rapid-onset, site-specific analgesia than standard intravenous analgesia alone. By reducing the use of GA drugs, the impairment of cognitive function in older patients can be modestly decreased. Local anesthetics have certain toxic effects on the central nervous system and can cause convulsions in severe cases, but no reports of causing POD were known, except for lidocaine and its patches, which had been shown to potentially induce delirium in a few case reports (Saravay et al., 1987; Byun et al., 2016).

Similarly, in elderly people with hip fractures, Gu et al. (2021) and Tang et al. (2021) compared multiple nerve blocks (NB) using ropivacaine and propofol-based IA, respectively. Interestingly, only the study by Gu et al. (2021) found a higher rate of early moderate delirium (24 h postoperatively) in the GA group than the NB group, with no significant between and within groups of severe POD. Bielka et al. (2021) used bupivacaine for PCB and sciatic nerve block in the aged undergoing hip fracture surgery. Although the results did not show any difference in the incidence of POD compared to SA or GA, the outcomes according to our NMA showed that PCB (SUCRA value = 42.2%) was preferred over other types of anesthesia methods (Supplementary Figure 8). Jankowski et al. (2003) reported that in outpatient knee arthroscopy, the analgesic effect of PCB was comparable to SA and superior to GA. Another trial compared the performance and complications of GA_PCB vs. GA_EA in hip surgery, showing that both performed similarly in terms of pain scores, but EA had noticeably more complications (Türker et al., 2003). Similarly, our NMA results suggest that with limited evidence, PCB may have a slight advantage in reducing POD and complications. The PCB is a form of NB commonly used as a component of multimodal anesthesia during hip and knee surgery. It appears to be superior to opioids for pain relief after hip surgery (Touray et al., 2008). Through the use of catheter techniques, PCB is used as an alternative to postoperative EA. However, we did not find relevant literature supporting the use of bupivacaine. On the contrary, some studies have reported that neurological complications were more common in the bupivacaine group compared to levobupivacaine. This issue remains to be explored by more prospective RCTs in the future.

When choosing the types of anesthesia, the anesthetics, effectiveness, and safety should not be overlooked. Postoperative nausea, vomiting, and hypotension are the two most common adverse effects of anesthesia. Risk factors associated with PONV include the use of opioids during and after surgery, the use of inhaled anesthetics and nitrous oxide, the duration of anesthesia, and so on (Rüsch et al., 2010). Some studies in our included RCTs reported the use of inhaled anesthetics, with Chen et al. (2001) and Li et al. (2021) reporting the administration of nitrous oxide during anesthesia maintenance. However, there were no statistically significant comparisons between anesthetic methods or anesthetics in terms of the occurrence of PONV. We speculate that it may be since almost all studies used opioid analgesics, such as fentanyl or morphine for intraoperative induction or postoperative analgesia, which is also one of the strong triggers of PONV (Horn et al., 2014). This may result in no difference in comparisons between groups. Postoperative hypotension is a common complication after anesthesia, especially in the SA and EA. According to our NMA conclusion, levobupivacaine-based SA was superior to bupivacaine-based SA in reducing postoperative hypotension in anesthetics comparison. It could be attributed to the fact that levobupivacaine pharmacologically has a higher safety margin and the sensory blockage time tends to be longer than bupivacaine (Casati and Putzu, 2005).

Some limitations of this systematic review and NMA cannot be ignored. First, limited by the number of published studies and inclusion criteria, only 19 RCTs were included in this systematic review and NMA. For the primary outcome, the incidence of POD, only seven RCTs were included in the anesthesia methods group, and 12 RCTs were in the anesthetics group. Second, in the primary outcome of this study, the comparison of overall anesthesia methods (GA, RA, and RA_GA) showed significantly local inconsistencies. To ensure the reliability of the results, we only performed a direct meta-analysis. Third, inconsistency in the type of surgery in elderly patients is a source of heterogeneity in this study. We did not conduct subgroup analysis or regressions test for different surgical methods because the results of the heterogeneity test were acceptable. Besides, this NMA conducted an indirect comparison of different anesthetics, but there were differences in patients’ perioperative medication and POD assessment methods between studies, which may lead to less reliable results. Finally, the methodological limitations of NMA cannot be ignored, which may lead to a different result by slight variations.

## Conclusion

In summary, this study based on mature methodology proved that GA and RA had no significant effect on POD, and there was no difference between anesthesia methods. In the results of the pairwise meta-analysis, except for RA with GA vs. GA, the rest of the results were not statistically different. Besides, PCB with bupivacaine may benefit to reduce POD incidence. However, clinical practitioners must choose the appropriate anesthesia method for individual patients.

## Data availability statement

The original contributions presented in this study are included in the article/Supplementary material, further inquiries can be directed to the corresponding author.

## Author contributions

XZ and YH: conceptualization, methodology, writing—original draft, data extraction, formal analysis, and writing—review and editing. YL: methodology, writing—original draft, and supervision. JL: formal analysis, writing—review and editing, and supervision. WM: conceptualization, methodology, supervision, and writing—review and editing. All authors contributed to the article and approved the submitted version.

## Supplementary material

Supplementary Figure 1 Risk of bias summary review authors’ judgments about each risk of bias item for each included study.

Supplementary Figure 2 Confidence of anesthesia methods groups’ data by CINEMA 2.0.0.

Supplementary Figure 3 Confidence of anesthetics groups’ data by CINEMA 2.0.0.

Supplementary Figure 4 Funnel plot of overall anesthesia groups’ POD incidence data.

Supplementary Figure 5 Funnel plot of anesthesia methods groups’ POD incidence data.

Supplementary Figure 6 Funnel plot of anesthetics groups’ POD incidence data.

Supplementary Figure 7 Plots of the surface under the cumulative ranking curves (SUCRA) for anesthesia methods in the incidence of POD.

Supplementary Figure 8 Plots of the surface under the cumulative ranking curves (SUCRA) for anesthetics in the incidence of POD.

## Abbreviations

PRISMA: Preferred Reporting Items for Systematic Reviews and Meta-analysis
CINeMA: Confidence in Network Meta-analysis
RCT: Randomized Controlled Trial
OR: Odds ratio
CI: confidence interval
NE: Not estimate
RA: regional anesthesia
GA: general anesthesia
SA: spinal anesthesia
CLSPB: Combined Lumbar-Sacral Plexus Block
PCB: Psoas compartment block
sevo: sevoflurane
prop: propofol
desf: desflurane
lido: lidocaine
bupi: bupivacaine
ropi: ropivacaine
keta: ketamine
IA: intravenous anesthesia
VA: ventilate anesthesia
NB: nerve block
SUCRA: surface under the cumulative ranking curve
RA_GA: regional anesthesia with general anesthesia
EA: epidural anesthesia
PONV: postoperative nausea and vomiting.
