# Supplementary figures and images for: The effects of anesthesia methods and anesthetics on postoperative delirium in the elderly patients: A systematic review and network meta-analysis
Source: Front Aging Neurosci. 2022 Nov 3;14:935716. doi: 10.3389/fnagi.2022.935716 (PMC9670185; doi:10.3389/fnagi.2022.935716)

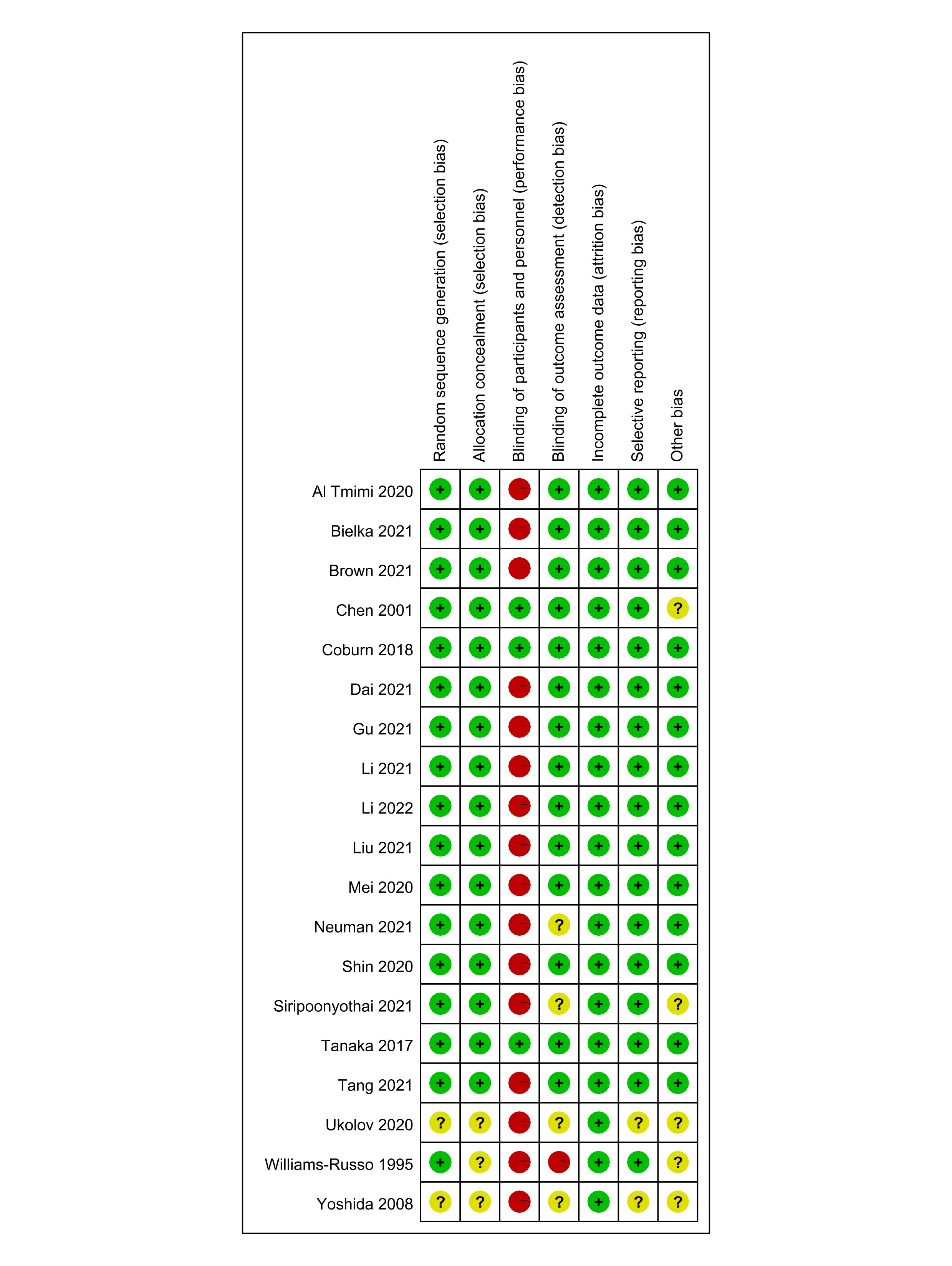

Supplement: Supplementary Figure 1 — Risk of bias summary review authors’ judgments about each risk of bias item for each included study. [file Image_1.JPEG]

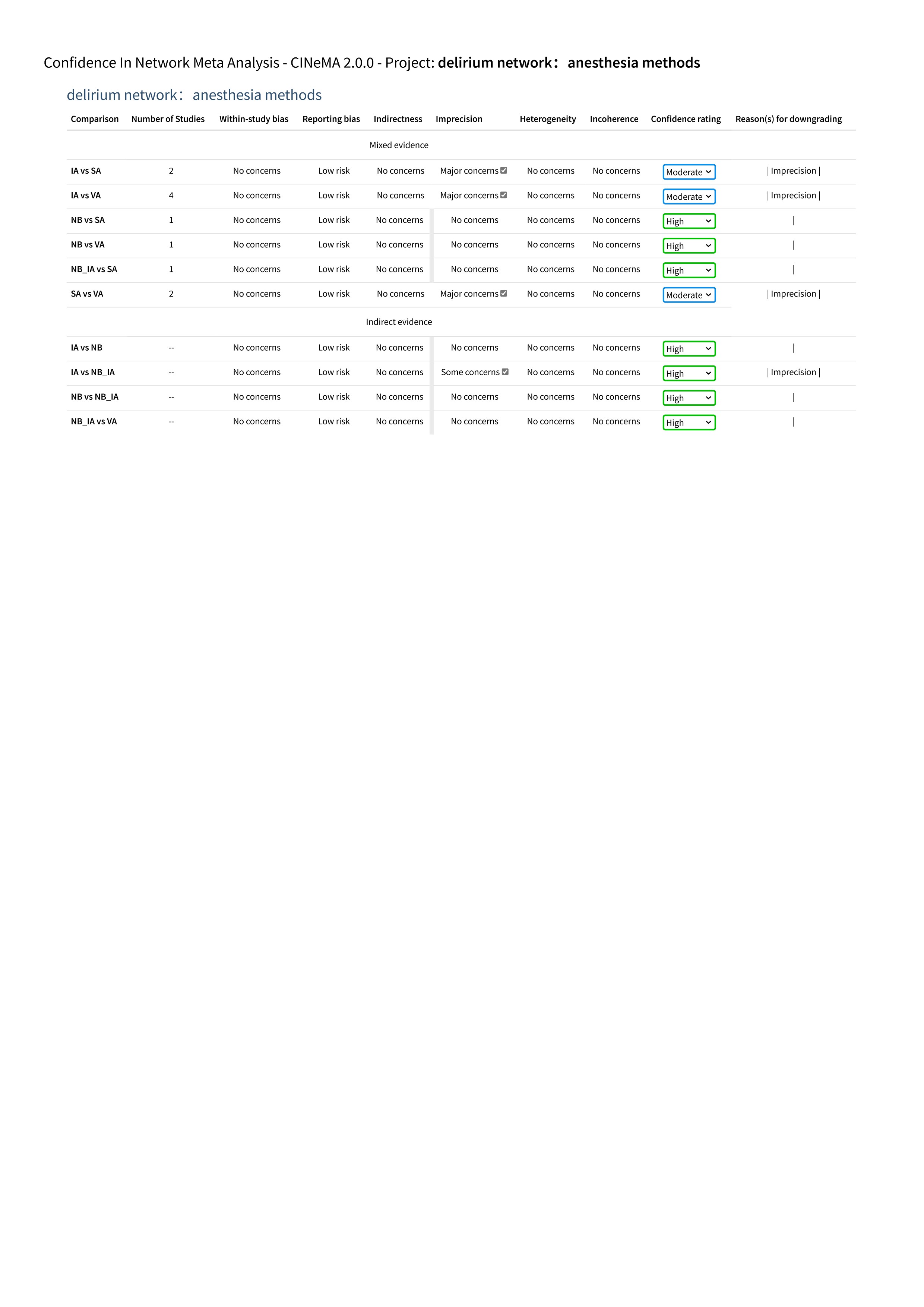

Supplement: Supplementary Figure 2 — Confidence of anesthesia methods groups’ data by CINEMA 2.0.0. [file Image_2.JPEG]

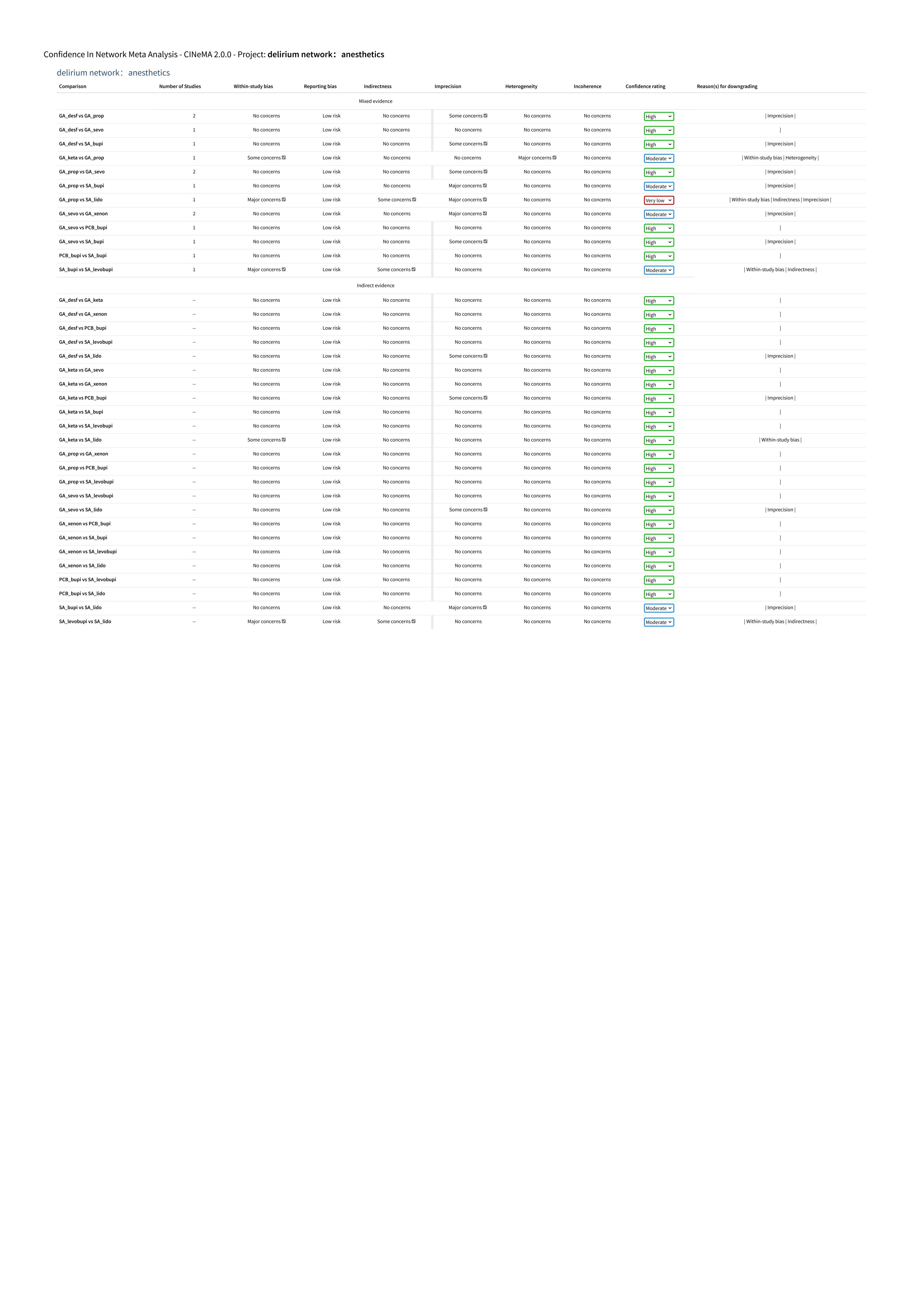

Supplement: Supplementary Figure 3 — Confidence of anesthetics groups’ data by CINEMA 2.0.0. [file Image_3.JPEG]

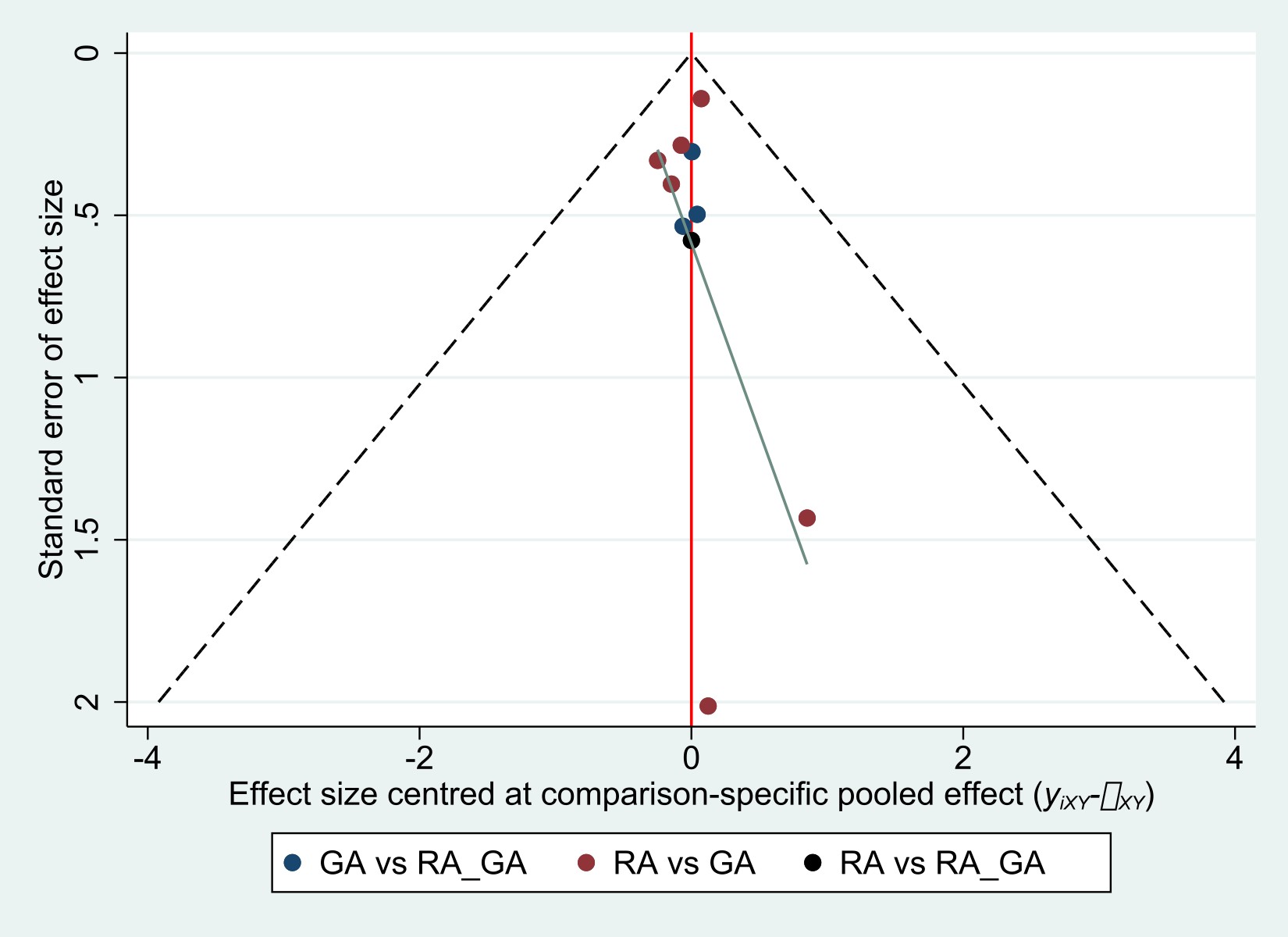

Supplement: Supplementary Figure 4 — Funnel plot of overall anesthesia groups’ POD incidence data. [file Image_4.JPEG]

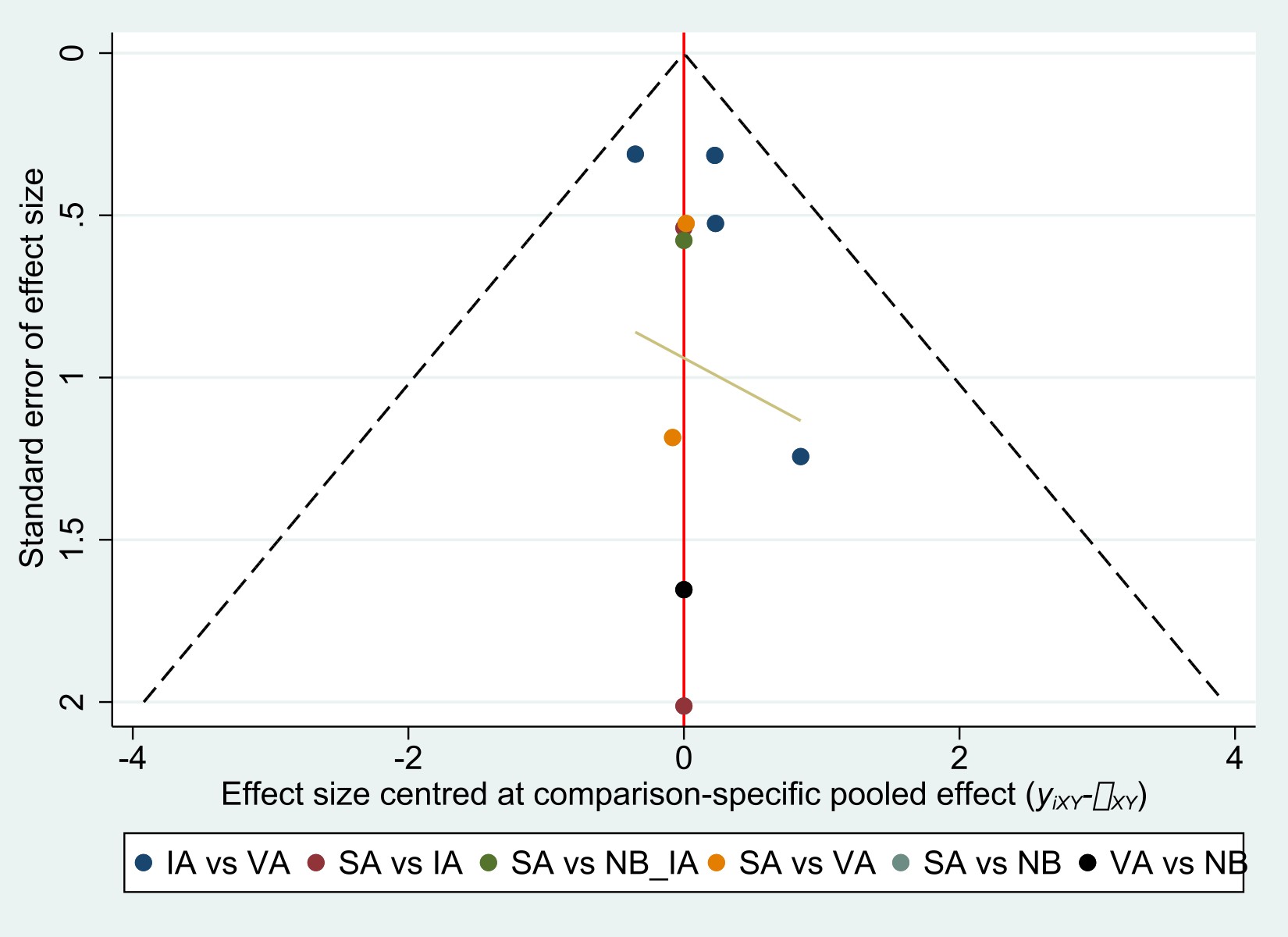

Supplement: Supplementary Figure 5 — Funnel plot of anesthesia methods groups’ POD incidence data. [file Image_5.JPEG]

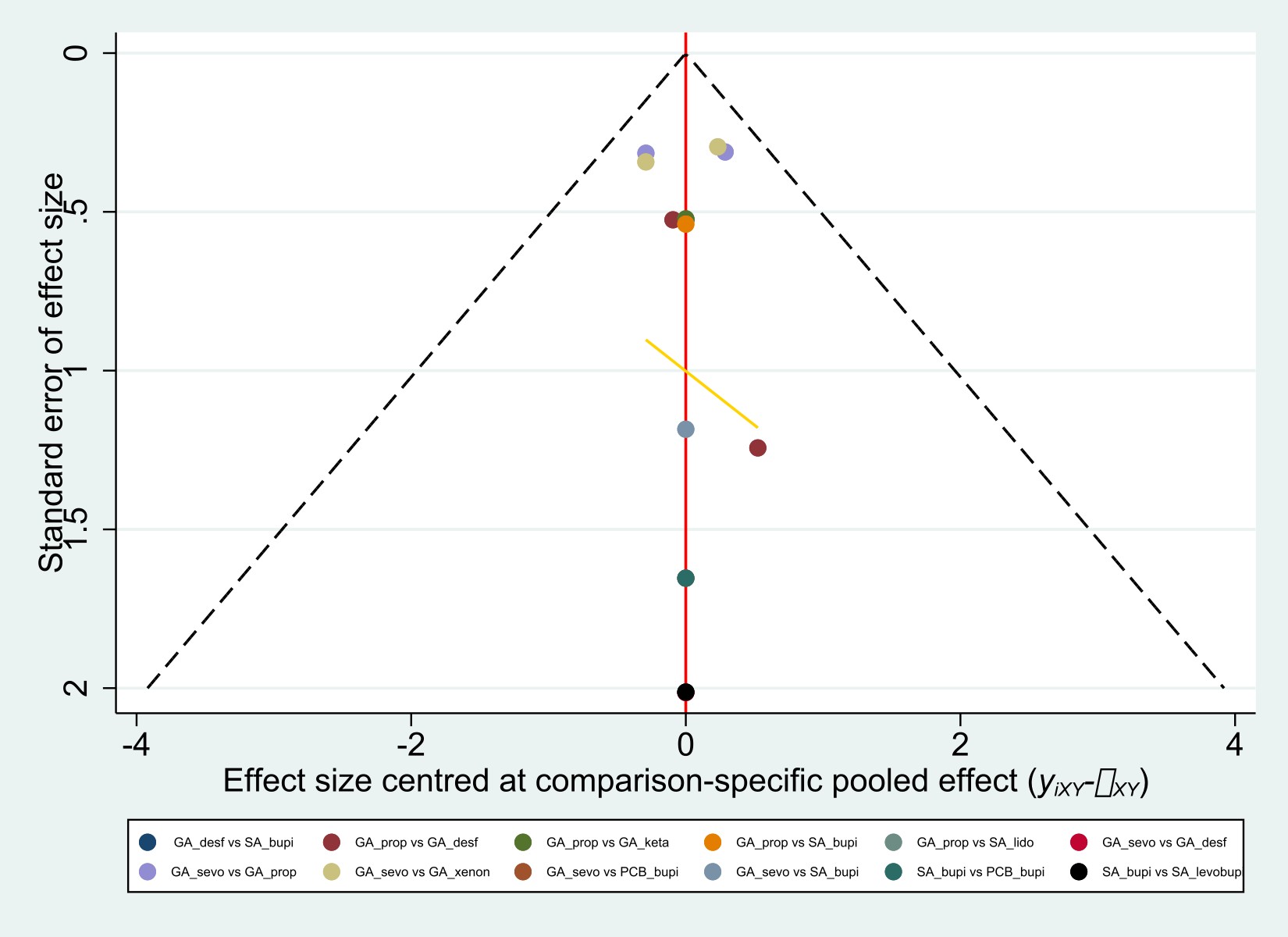

Supplement: Supplementary Figure 6 — Funnel plot of anesthetics groups’ POD incidence data. [file Image_6.JPEG]

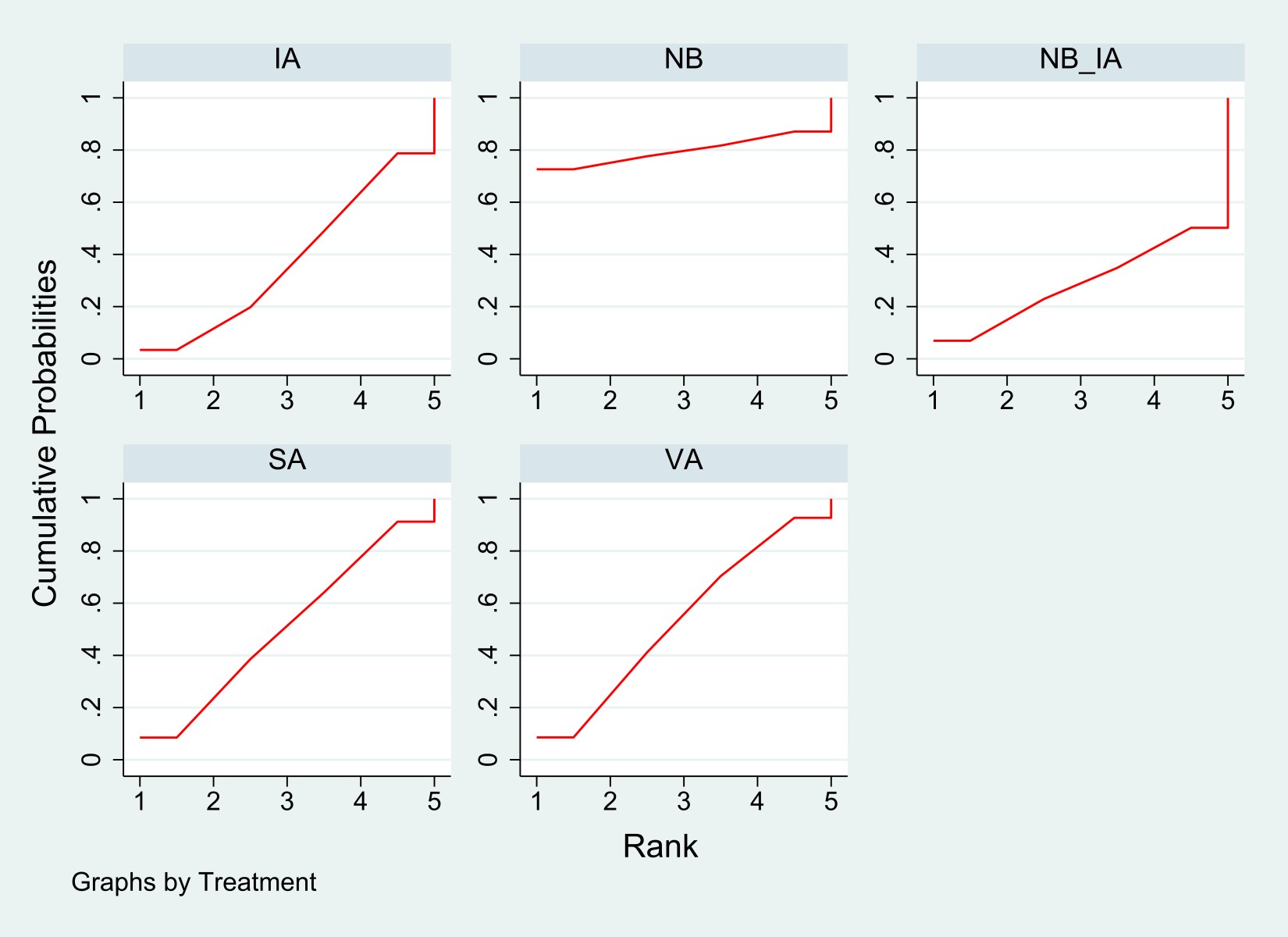

Supplement: Supplementary Figure 7 — Plots of the surface under the cumulative ranking curves (SUCRA) for anesthesia methods in the incidence of POD. [file Image_7.JPEG]

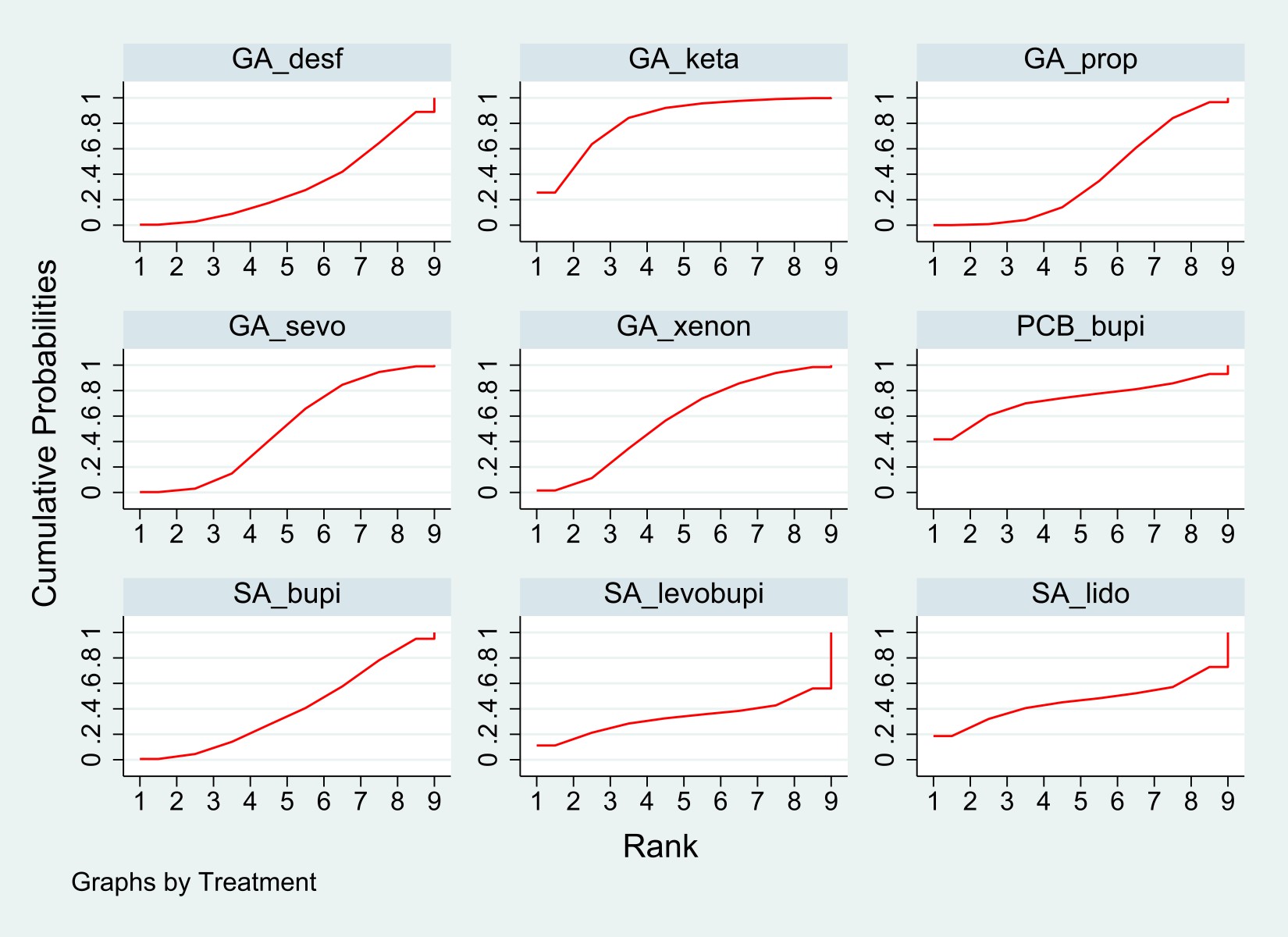

Supplement: Supplementary Figure 8 — Plots of the surface under the cumulative ranking curves (SUCRA) for anesthetics in the incidence of POD. [file Image_8.JPEG]
